# PHYSICAL ACTIVITY IN EARLY AND MIDDLE ADULTHOOD PREDICTS LATER-LIFE MEMORY TRAJECTORIES VIA HEALTH PATHWAYS

**DOI:** 10.1093/geroni/igz038.3288

**Published:** 2019-11-08

**Authors:** Zarina Kraal, Laura B Zahodne

**Affiliations:** University of Michigan, Ann Arbor, Michigan, United States

## Abstract

Physical inactivity measured during late-life is a modifiable risk factor for dementia, but many studies use concurrent assessments with limited longitudinal follow-up. Less is known regarding life course exposure to physical inactivity. Physical activity patterns at different ages may make independent contributions to dementia risk, which would point to multiple critical periods for intervention. Using Health and Retirement Study Life History Mail Survey data (N=4,396), latent growth curves tested whether retrospectively-reported activity in early (18-29 years) and middle (40-49 years) adulthood predicted later-life memory trajectories over 18 years (mean age at study entry = 60.56 ± 5.44; mean follow-up = 13.27 ± 4.03 years). Total metabolic equivalents were computed from reports of moderate and vigorous physical activity. Biennial memory performance was modeled from study entry (between 1996 and 2014) to 2014. Self-reported physical and mental health at study entry were modeled as independent mediators. Models were adjusted for age at study entry, sex, education, race, ethnicity, childhood socio-economic status, year of study entry, and year of mail survey enrollment. More physical activity at ages 18-29 and 40-49 were independently associated with better initial memory, but not subsequent memory change. The association between physical activity at ages 40-49 and initial memory was partially mediated by better mental and physical health. These observational results support the possibility that physical activity interventions during multiple stages of the adult life course might be effective at lowering dementia risk. In particular, mid-life physical activity may have broad effects on later mental, physical, and cognitive health.

